# Δ^8^-THC Protects against Amyloid Beta Toxicity Modulating ER Stress In Vitro: A Transcriptomic Analysis

**DOI:** 10.3390/ijms24076598

**Published:** 2023-04-02

**Authors:** Agnese Gugliandolo, Santino Blando, Stefano Salamone, Diego Caprioglio, Federica Pollastro, Emanuela Mazzon, Luigi Chiricosta

**Affiliations:** 1 IRCCS Centro Neurolesi “Bonino-Pulejo”, Via Provinciale Palermo, Contrada Casazza, 98124 Messina, Italy; 2 Department of Pharmaceutical Sciences, University of Eastern Piedmont, Largo Donegani 2, 28100 Novara, Italy; 3 PlantaChem Srls, Via Amico Canobio 4/6, 28100 Novara, Italy

**Keywords:** Alzheimer’s disease, Δ^8^-THC, unfolded protein response, neuronal apoptosis

## Abstract

Alzheimer’s disease (AD) represents the most common form of dementia, characterized by amyloid β (Aβ) plaques and neurofibrillary tangles (NFTs). It is characterized by neuroinflammation, the accumulation of misfolded protein, ER stress and neuronal apoptosis. It is of main importance to find new therapeutic strategies because AD prevalence is increasing worldwide. Cannabinoids are arising as promising neuroprotective phytocompounds. In this study, we evaluated the neuroprotective potential of Δ^8^-THC pretreatment in an in vitro model of AD through transcriptomic analysis. We found that Δ^8^-THC pretreatment restored the loss of cell viability in retinoic acid-differentiated neuroblastoma SH-SY5Y cells treated with Aβ_1-42_. Moreover, the transcriptomic analysis provided evidence that the enriched biological processes of gene ontology were related to ER functions and proteostasis. In particular, Aβ_1-42_ upregulated genes involved in ER stress and unfolded protein response, leading to apoptosis as demonstrated by the increase in Bax and the decrease in Bcl-2 both at gene and protein expression levels. Moreover, genes involved in protein folding and degradation were also deregulated. On the contrary, Δ^8^-THC pretreatment reduced ER stress and, as a consequence, neuronal apoptosis. Then, the results demonstrated that Δ^8^-THC might represent a new neuroprotective agent in AD.

## 1. Introduction

Alzheimer’s disease (AD) is the most common neurodegenerative disorder and represents the most frequent cause of dementia, affecting more than 50 million people worldwide [1,2]. Considering that the strongest risk factor for AD is aging and that life expectancy is gradually increasing, the number of AD patients is also progressively rising [3,4].

Even though AD is mainly sporadic and shows a late onset (>65 years of age), there are rare cases in which the disease is associated with autosomal dominant inheritance and usually develops earlier (between 24 and 60 years of age) [5]. More than 300 pathogenic mutations in presenilin 1 (PSEN1), presenilin 2 (PSEN2), and amyloid precursor protein (APP) genes have been identified in familial AD cases [5]. Instead, allelic variation in the apolipoprotein E (APOE) gene represents a major genetic risk factor for sporadic AD [6].

The most important neuropathological features of AD are β-amyloid (Aβ)-containing extracellular plaques and tau-containing intracellular neurofibrillary tangles [7,8]. In addition, AD is characterized by the atrophy of the cerebral cortex and by the loss of basal forebrain cholinergic neurons [9]. 

It is known that AD patients generally manifest prominent amnestic cognitive impairment; however, non-amnestic cognitive impairment is less frequent [8]. AD symptoms can vary depending on the stage of the disease and AD can be classified as preclinical or presymptomatic, mild, and dementia-stage on the basis of the degree of cognitive impairment [4]. 

Currently, the diagnostic methods for AD primarily rely on neurocognitive tests, brain imaging techniques and cerebrospinal fluid assays [10].

The “amyloid cascade hypothesis” is one of the most important models for the pathogenesis of AD and suggests that the deposition and accumulation of Aβ cause the formation of amyloid plaques, leading to neuronal and synaptic toxicity in the brain. The resulting neuronal damage can lead to memory and cognition dysfunctions [11]. Although the mechanisms implicated in Aβ-induced neurotoxicity are still not completely clarified, it has been suggested that different pathways, including oxidative stress, microglial activation and apoptosis, can be involved [12]. In addition, interestingly, it has been shown that endoplasmic reticulum (ER) stress can be implicated in AD [12]. In particular, it is known that ER is responsible for the biosynthesis of proteins, including the post-translational modification, folding and assembly of newly synthesized proteins, and it has been suggested that the accumulation of insoluble Aβ-peptides could alter ER homeostasis, leading to ER stress and thus activating the unfolded protein response (UPR) [12,13]. Although, at first, UPR aims to restore the normal function of ER, prolonged stress can lead to the activation of apoptotic factors [12]. Considering that it has been shown that ER stress can be involved in Aβ-induced apoptosis, it should be noted that the inhibition of ER stress could exert beneficial effects [12].

Currently, there are no efficacious treatments that are able to reverse or delay the progression of AD [14]. The drugs approved by the US Food and Drug Administration (FDA) for the treatment of AD include the cholinesterase inhibitors donepezil, galantamine and rivastigmine; the N-methyl-D-aspartate (NMDA) receptor antagonist memantine; a combination of memantine and donepezil; the monoclonal antibodies targeting Aβ aducanumab and lecanemab [14,15,16,17,18]. 

Of note, the possible therapeutic use of cannabinoids in AD has been recently investigated [19]. Studies in rodent models with AD have highlighted the promising effects of cannabinoids in decreasing amyloid plaque deposition and inducing hippocampal neurogenesis, whereas clinical studies have shown the beneficial effects of cannabinoid treatment on AD symptoms [20]. 

In particular, it has been suggested that delta8-tetrahydrocannabinol (∆^8^-THC), a cannabinoid that is a structural isomer of a widely known active ingredient in cannabis delta9-tetrahydrocannabinol (Δ^9^-THC), could exert interesting pharmacological effects [21,22]. It is considered to have fewer potent psychoactive properties than Δ^9^-THC [23]. ∆^8^-THC is a partial agonist of the cannabinoid CB1 receptor, while it has also been reported to be an agonist or inverse agonist at the CB2 receptor. Cannabinoids can also interact with other receptors, such as PPARγ and GRP55, but there are no data about Δ^8^-THC effects on these receptors [22]. ∆^8^-THC use may be associated with decreased chemotherapy side effects, analgesic effects, decreased seizure activity, lower intra-ocular eye pressure, decreased cancer cell proliferation, decreased depressive symptoms and decreased nicotine use and withdrawal [23]. In addition, it has been shown that Δ^8^-THC exerts moderate inhibitory activity against acetylcholinesterase and butyrylcholinesterase [24]. This is of particular interest, considering that it is known that the enhancement of cholinergic neurotransmission through cholinesterase inhibitors is the leading therapeutic option for treating the cognitive and behavioral symptoms of the early and late stages of AD [25].

In this study, we evaluated the neuroprotective potential of ∆^8^-THC in an in vitro model of AD using Next Generation Sequencing (NGS). With this aim, we pretreated retinoic acid (RA)-differentiated SH-SY5Y neuroblastoma cells with ∆^8^-THC and exposed them to Aβ_1-42_. At the end of the treatment, we performed a transcriptomic analysis in order to evaluate whether ∆^8^-THC could modulate signaling pathways leading to protective effects. 

## 2. Results

### 2.1. Δ^8^-THC Counteracted the Aβ_1-42_-Induced Loss of Cell Viability

RA-differentiated SH-SY5Y were pretreated with different doses of Δ^8^-THC for 24 h and, after, were treated with 10 µM Aβ_1-42_ for another 24 h. Using the MTT assay, we evaluated if Δ^8^-THC exerted toxicity in the range of the doses tested (5–20 µM) and if it was able to counteract Aβ_1-42_ toxicity.

**Figure 1:**
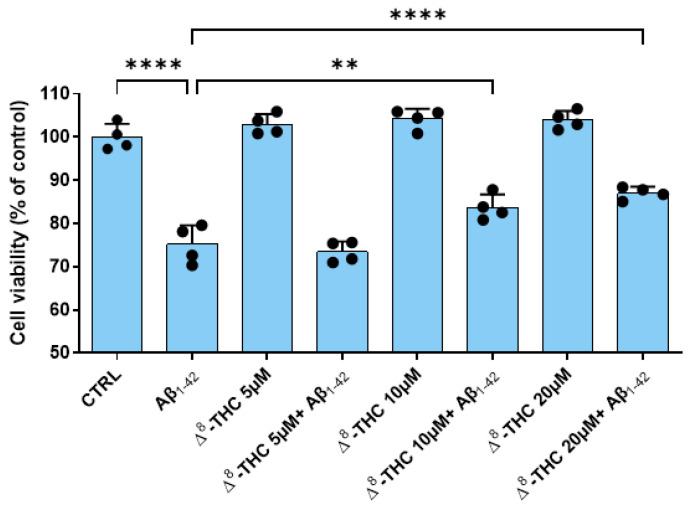
Cell viability after Aβ_1-42_ and Δ^8^-THC treatment. The treatment with 10 µM Aβ_1-42_ reduced cell viability of RA-differentiated SH-SY5Y cells, but Δ^8^-THC pretreatment at the concentrations 10 and 20 µM was able to restore cell viability. N = 4 independent experiments. The results are expressed by mean ± standard deviation (SD). ** *p* < 0.01; **** *p* < 0.0001.

The MTT assay results demonstrated that 10 µM Aβ_1-42_ treatment reduced the cell viability of RA-differentiated SH-SY5Y cells. Δ^8^-THC was not cytotoxic at all the concentrations tested. However, 5 µM Δ^8^-THC was not able to counteract Aβ_1-42_-reduced cell viability. On the contrary, both 10 and 20 µM Δ^8^-THC were able to restore the cell viability of RA-differentiated SH-SY5Y cells after 10 µM Aβ_1-42_ treatment (Figure 1). Transcriptomic analysis was carried out using the concentration 20 µM Δ^8^-THC.

### 2.2. Transcriptomic Analysis Revealed That Δ^8^-THC Counteracted the Aβ_1-42_-Induced ER Stress

**Figure 2:**
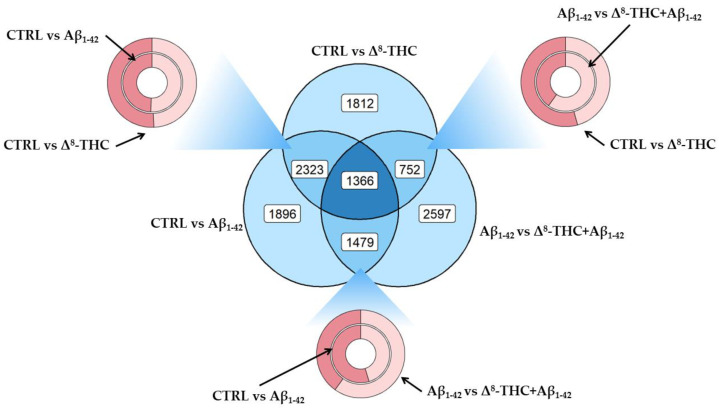
DEGs distribution between CTRL vs. Aβ_1-42,_ CTRL vs. Δ^8^-THC groups or Aβ_1-42_ vs. Δ^8^-THC + Aβ_1-42_. Venn diagram in the center of the plot highlights the amount of DEGs found exclusively in each comparison (outer circles), how many DEGs found in two groups but not in the other (intersection of two circles) or how many DEGs were found in each comparison (center of the diagram). Each donut plot highlights, in turn, the intersection of two comparisons showing in dark red the upregulated and in the light red the downregulated DEGs.

In order to evaluate the differential pattern of gene expression in RA-differentiated SH-SY5Y treated with 20 µM Δ^8^-THC and 10 µM Aβ_1-42_, we performed NGS transcriptomic analysis. The aim was to evaluate the pathways associated with the protective effects exerted by Δ^8^-THC. Figure 2 represents the distribution of differentially expressed genes (DEGs) between control against Aβ_1-42_ (CTRL vs. Aβ_1-42_), control against Δ^8^-THC (CTRL vs. Δ^8^-THC), or Aβ_1-42_ against Δ^8^-THC + Aβ_1-42_ groups (Aβ_1-42_ vs. Δ^8^-THC + Aβ_1-42_). In the blue section, we highlighted how many DEGs were exclusively deregulated in each comparison (1896 in CTRL vs. Aβ_1-42_, 1812 in CTRL vs. Δ^8^-THC and 2597 in Aβ_1-42_ vs. Δ^8^-THC + Aβ_1-42_), between two comparisons and not in the last one (2323 not in Aβ_1-42_ vs. Δ^8^-THC + Aβ_1-42_, 752 not in CTRL vs. Aβ_1-42_, 1479 not in CTRL vs. Δ^8^-THC) or deregulated in all the comparisons (1366 DEGs). On the other hand, the three donuts show how many upregulated (dark red) or downregulated (light red) DEGs were found between each comparison and, in particular, between CTRL vs. Aβ_1-42_ and CTRL vs. Δ^8^-THC in the left-top section, CTRL vs. Aβ_1-42_ and Aβ_1-42_ vs. Δ^8^-THC + Aβ_1-42_ in the bottom section and CTRL vs. Δ^8^-THC and Aβ_1-42_ vs. Δ^8^-THC + Aβ_1-42_ in the right-top section.

**Figure 3:**
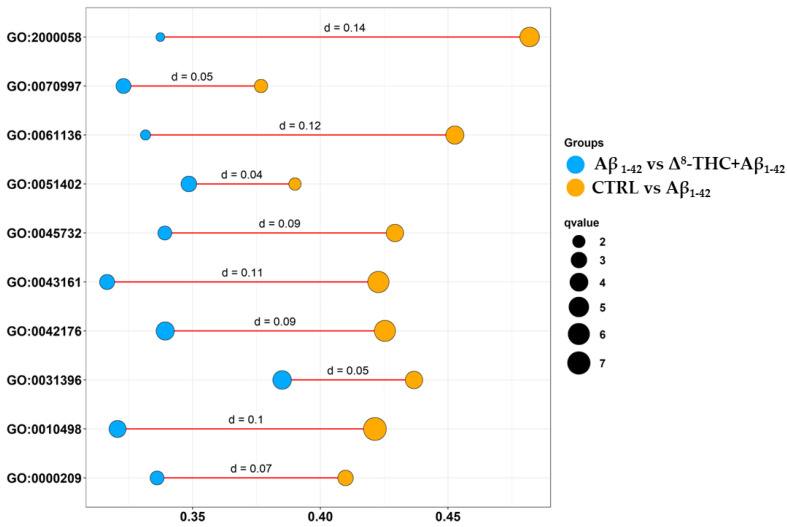
Bubbleplot of biological process terms enriched in gene ontology between CTRL vs. Aβ_1-42_ and Aβ_1-42_ vs. Δ^8^-THC + Aβ_1-42_ groups. For each ontology reported on the *y* axis, a bubble for the CTRL vs. Aβ_1-42_ (orange) and one for the Aβ_1-42_ vs. Δ^8^-THC + Aβ_1-42_ (light blue) groups were plotted. The position of the bubble in the *x* axis shows the number of DEGs in the ontology (the more on the right, the higher the number of DEGs). The number of DEGs was normalized over the number of genes included in the ontology term itself so that the terms were comparable to each other. The size of the bubble is a score given by −log(q-value).

We then enriched DEGs both in CTRL vs. Aβ_1-42_ and Aβ_1-42_ vs. Δ^8^-THC + Aβ_1-42_ in order to evaluate if Δ^8^-THC was able to exert protective effects through the modulation of processes affected by Aβ_1-42_. Specifically, we enriched DEGs for the biological process terms of gene ontology (GO) that revealed 665 terms in CTRL vs. Aβ_1-42_ and 488 terms in Aβ_1-42_ vs. Δ^8^-THC + Aβ_1-42_ groups. The inspection of the biological process terms commonly enriched in the two analyses revealed 321 terms. In this line, we depicted the bubble plot in Figure 3 that shows how DEGs were enriched for each ontology in the CTRL vs. Aβ_1-42_ (orange) or Aβ_1-42_ vs. Δ^8^-THC + Aβ_1-42_ (light blue) groups. They demonstrated the “regulation of ubiquitin-dependent protein catabolic process” (GO:2000058), “neuron death” (GO:0070997), the “regulation of proteasomal protein catabolic process” (GO:0061136), “neuron apoptotic process” (GO:0051402), “positive regulation of protein catabolic process” (GO:0045732), “proteasome-mediated ubiquitin-dependent protein catabolic process” (GO:0043161), “regulation of protein catabolic process” (GO:0042176), “regulation of protein ubiquitination” (GO:0031396), “proteasomal protein catabolic process” (GO:0010498), “protein polyubiquitination” (GO:0000209). Interestingly, all the aforementioned ontologies included a higher number of DEGs in CTRL vs. Aβ_1-42_ than in Aβ_1-42_ vs. Δ^8^-THC + Aβ_1-42_ groups. 

Given that significantly enriched GO are related to ER functions and proteostasis, we focused on related DEGs looking at KEGG pathways “Alzheimer disease” (hsa05010) and “protein processing in endoplasmic reticulum” (hsa04141). In Table 1, we report the common DEGs, altered in both CTRL vs. Aβ_1-42_ and Aβ_1-42_ vs. Δ^8^-THC *+* Aβ_1-42_. In the supplementary Table S1, we report all the inspected DEGs. 

### 2.3. Δ^8^-THC Restored the Protein Levels of Bax and Bcl-2

**Figure 4:**
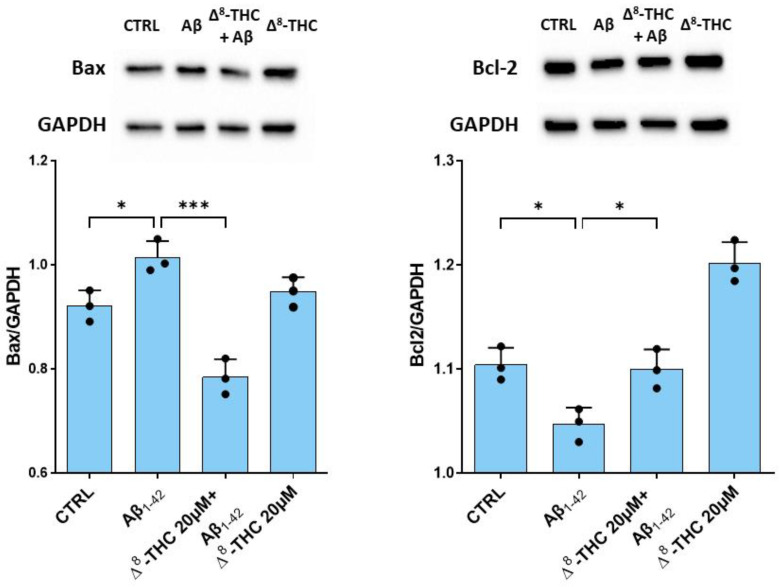
Western blot for Bax and Bcl-2. Aβ_1-42_ treatment caused an increase in Bax and a reduction in Bcl-2 protein levels. Δ^8^-THC treatment restored protein levels of Bax and Bcl-2. N = 3 independent experiments. The results are expressed by mean ± standard deviation (SD). * *p* < 0.05; *** *p* < 0.001.

In order to evaluate the effects of Δ^8^-THC on Aβ_1-42_-induced apoptosis, we evaluated the levels of Bax and Bcl-2. Western blot analysis evidenced a significant increase in Bax in RA-differentiated SH-SY5Y treated with 10 µM Aβ_1-42_. Pre-treatment with 20 µM Δ^8^-THC was able to reduce Bax protein levels. On the contrary, Bcl-2 protein levels were decreased in Aβ_1-42_ treated cells, while Δ^8^-THC restored its levels (Figure 4). Δ^8^-THC treated RA-differentiated SH-SY5Y showed a level of Bax similar to the control, while Bcl-2 increased.

## 3. Discussion

The prevalence of dementia is increasing, and it is expected that about 113 million will be affected in 2050 worldwide [26]. Given that AD is the prevalent form of dementia, it is of main importance to find new therapeutic strategies. Cannabinoids seem promising for neuroprotective treatments. Some of them have been reported to improve cognitive functions and reduce Aβ [20]. Δ^8^-THC is present in a very low quantity in plants, and it is mainly produced by cannabidiol. Δ^8^-THC is a structural isomer of the Δ^9^-THC, showing a double bond between carbon atoms 8 and 9 rather than carbon atoms 9 and 10. Δ^9^-THC is responsible for the psychoactive properties of cannabis, such as alterations in mood, perception and cognition. Δ^9^-THC is one of the cannabinoids most studied, but Δ^8^-THC has also been attracting attention for the better thermodynamic stability in comparison to Δ^9^-THC. The two compounds showed similar pharmacokinetics and pharmacodynamics. Both Δ^8^-THC and Δ^9^-THC are partial agonists of the cannabinoid receptor CB1, but Δ^8^-THC showed a lower affinity. Given that the psychoactive effects of Δ^9^-THC depend on the CB1 receptor, Δ^8^-THC has a lower psychotropic potency. Both Δ^8^-THC and Δ^9^-THC were reported to act as agonists or inverse agonists at the CB2 receptor [22]. A survey of consumers highlighted that Δ^8^-THC might exert the benefits of Δ^9^-THC with lower risks [27]. 

Some cannabinoids have already shown protective effects in both in vitro and in vivo AD models. Δ^9^-THC was shown to lower Aβ levels in an in vitro AD model in a dose-dependent manner, directly binding to the Aβ peptide and inhibiting its aggregation [28]. Moreover, it can alleviate cognitive impairments and reduce inflammatory markers, the numbers of Aβ plaques and degenerated neurons in AD mice [29,30,31]. Additionally, other agonists of CB1 and CB2 receptors were tested in AD models. ACEA, a CB1 receptor agonist, exerted a strong neuroprotective action against Aβ toxicity in vitro and in vivo [32,33]. Additionally, CB2 agonists, such as JWH-133, showed neuroprotective effects in AD models, reducing inflammation, Aβ plaque and deposition, increasing Aβ clearance and improving cognitive performance [34]. CP55940, an agonist of CB1 and CB2, restored mitochondrial membrane potential and reactive oxygen species and reduced extracellular Aβ [35].

However, the potential neuroprotective effects of Δ^8^-THC have not been investigated yet. To our knowledge, this is the first study that has investigated Δ^8^-THC effects in an in vitro model of AD. 

In this study, we found that Δ^8^-THC showed no cytotoxicity at all the doses tested. Interestingly, it was able at doses 10 and 20 µM to restore the loss of cell viability induced by Aβ_1-42_. The dose of 20 µM Δ^8^-THC was used for other experiments. 

Transcriptomic analysis and GO evaluations evidenced the enrichment of biological processes related to proteostasis and neuronal apoptosis. Then, Δ^8^-THC could modulate the pathways involved in proteostasis to exert protective effects. For this reason, we focused on DEGs related to ER functions and proteostasis, looking at the KEGG pathways for “Alzheimer’s disease” (hsa05010) and “protein processing in endoplasmic reticulum” (hsa04141). 

ER plays important roles in protein biosynthesis and in their quality control. In some cases, the maintenance of cellular homeostasis is not possible, causing a reduction in the protein folding capacity of ER and leading to the accumulation of misfolded/unfolded proteins in ER. This process caused the disruption of cellular homeostasis, inducing ER stress. AD is characterized by protein misfolding and aggregation and Aβ accumulation, which are all events that trigger ER stress [36]. 

Treatment with Aβ_1-42_ also altered the expression of several chaperones. Chaperones are a functionally related group of proteins that assist protein folding both in physiological and stress conditions. Among chaperones, heat shock proteins (HSPs) are well known. HSPs have a role in all the phases of proteostasis; they participate in folding, protein synthesis and degradation. Their levels increase during stress exposure, helping to prevent conformational changes and the aggregation of misfolded proteins [37]. We found that several HSPs were dysregulated by Aβ_1-42_ treatment. Aβ_1-42_ treatment increased the expression of several members of the HSP40 family (*DNAJC1*, *DNAJC10*, *DNAJC3*), HSP70 family (*HSPA1A* and *HSPA1L*) and HSP90 family (*HSP90AA1* and *HSP90AB1*). HSPs also played a role in the degradation of proteins by the proteasome. The genes encoding for these HSPs, such as *DNAJA2* and *DNAJB12,* were downregulated by Aβ_1-42_ treatment. 

We also found the upregulation of *HSP90B1,* encoding for GRP94, after Aβ_1-42_ treatment. GRP94 is a chaperone that directs the folding and/or assembly of proteins. Moreover, GRP94 is one of the few major luminal calcium-binding proteins. It seems to have a role in ER-associated degradation (ERAD) to distinguish misfolded proteins and target them for degradation [38]. Δ^8^-THC reduced the levels of *HSP90B1, DNAJC1* and *HSP90AB1* while increasing *DNAJA2* expression in cells treated with Aβ_1-42_.

The excess of misfolded proteins induces ER stress. In order to counteract ER stress, cells activate UPR. The UPR starts as cell-protective cascades, with the aim of reducing the ER load of unfolded proteins through the inhibition of protein synthesis and the upregulation of protein folding and degradation. However, prolonged UPR finally leads to cell death. The UPR signaling involves three sensor proteins, which are PERK, ATF6, and IRE1. Abnormal levels of these effectors of UPR were reported in AD brains [39]. We found the upregulation of PERK (*EIFAK2* and *EIFAK4*), ATF6 (*ATF6*) and IRE1 (*ERN1*) in cells treated with Aβ_1-42_. PERK activation is reported in AD and is associated with neurodegeneration and memory deficits [40]. Additionally, IRE1 activation is known to participate in AD pathogenesis and to be positively correlated with the progression of AD [41]. IRE1 activation is also associated with the induction of apoptosis [42]. Active ATF6 has also been shown in AD models [42]. We also found the downregulation of *WFS1*, which influences ER stress, to negatively regulate ATF6α [43]. Interestingly, it was found that the protein level of WFS1 and the number of WFS1+ neurons decreased in both AD-like mouse model brains and human post-mortem AD [44]. In particular, WFS1 deficiency was linked with increased tau pathology and neurodegeneration. WFS1 deficiency may induce chronic ER stress and affect the degradation and clearance of tau aggregates [45]. Interestingly, Δ^8^-THC pre-treatment reduced the expression of the genes encoding for PERK, ATF6 and IRE1.

Misfolded/unfolded proteins are eliminated by the proteasome through ERAD. ERAD can be divided into four steps that are substrate recognition, dislocation across the membrane, ubiquitination and degradation by the proteasome [46]. The data suggest alterations of ERAD in AD [47,48,49,50]. In this study, we found a dysregulation of genes involved in the ERAD process. Specifically, genes involved in the phase of the recognition of unfolded protein were upregulated by Aβ_1-42_. We found the upregulation of genes encoding for ERManI (*MAN1A2* and *MAN1B1*). Removal of mannose residues is a critical process in targeting misfolded glycoproteins for degradation. This removal is operated by ERManI together with EDEM [51,52]. This trimming permits misfolded glycoproteins to be bound to OS-9 and XTP3-B, which target them to ERAD. OS-9 is upregulated in response to ER stress and is required for the ubiquitination of ERAD substrates, suggesting that it may help transfer misfolded proteins to ubiquitination machinery [53]. OS9 and XTP3B redundantly promote glycoprotein degradation, but XTP3B inhibits the degradation of non-glycosylated proteins, while OS9 antagonizes this inhibition [54]. After treatment with Aβ_1-42,_ we also found the upregulation of OS-9 and XTP3B (*ERLEC1*). We also found the downregulation of *EDEM1*. It can modulate APP metabolism, and its overexpression is associated with a decrease in Aβ secretion [55]. Interestingly, *EDEM1* can be upregulated by Δ^8^-THC pre-treatment. Δ^8^-THC pre-treatment also reduced OS-9 and *MAN1B1.*

We also found deregulation in the genes encoding for PDI (*PDIA6*, *P4HB*, *TXNDC5*) and ERO (*ERO1A*). Aβ_1-42_ increased the genes encoding for ERO while reducing the expression of those encoding for PDI. In the oxidizing environment of the ER, unfolded proteins interact with PDI and undergo oxidative protein folding. In this way, misfolded substrate proteins can be reduced and refolded or isomerized to the appropriate native protein conformation. Misfolded proteins are reduced and isomerized by PDI and converted to their appropriate native conformation. Then, reduced PDI is reoxidized by ERO1. Δ^8^-THC pre-treatment reduced ERO (*ERO1B*) while also increasing PDI (*ERP29* and *TXNDC5*).

On the contrary, the genes involved in the processes of translocation, ubiquitination and degradation were mainly downregulated by Aβ_1-42_. Bap31, encoded by *BCAP31* which we found downregulated, have several roles in ER homeostasis: membrane protein chaperone, quality control, and it is involved in ER stress and ERAD [56]. Its deficiency was associated with the formation of Aβ plaques in a murine AD model [57]. P97, encoded by *VCP*, also plays a critical role in protein dislocation in ERAD [46]; it is involved in aggregates clearance, and, indeed, its knockdown delayed the elimination of ubiquitin-positive aggregates [58]. We found the downregulation of *VCP* after Aβ_1-42_ treatment. 

The poly-ubiquitination of proteins is fundamental for their degradation by the proteasome, and different enzymes are required: an E1 activating enzyme activates ubiquitin in an ATP-dependent manner, an E2 ubiquitin-conjugating enzyme (Ubc), and an E3 ubiquitin-protein ligases that mediate the transfer of ubiquitin from the Ubc enzyme onto the target substrate. We found a downregulation in ubiquitin ligase complex subunits after treatment with Aβ_1-42_, such as *UBE2G2* and others (*RBX1*, *UBE2J1*, *UBQLN1*, *UBQLN2*, *UBQLN4*, *UBXN6*, *UBXN8*). UBE2G2 was shown to be critically important for degradation through the ERAD of multiple substrates [59]. Additionally, *FBXO6* was downregulated; it is a functional E3 ubiquitin ligase that plays a critical role in inhibiting ER stress-induced apoptosis [60].

Δ^8^-THC pretreatment was able to reverse the alterations of the gene expression induced by Aβ_1-42_. In particular, the genes involved in protein targeting (*EDEM3*, *MAN1B1*) were downregulated, suggesting that there was less need for proteins involved in unfolded protein recognition. *SEC61A1* and *SEC61B* were downregulated. The Sec61, which interacts with TRAP [61], encoded by *SSR3,* which was upregulated, mediates protein import into the ER and is also a candidate channel for the dislocation of ERAD substrates [62,63]. The expression of the genes involved in protein dislocation and ubiquitination increases (*UBE2G2*, *SELENOS*, *TRAM1*, *UBE2J1*, *UBXN6*, *UBXN8*, *FBXO6*). Then, if needed, misfolded/unfolded proteins can be dislocated to the cytosol and ubiquitinated. 

Interestingly, Δ^8^-THC reduced the expression of *HERPUD1*, encoding for HERP. HERP was reported to be involved in Aβ accumulation, including the formation of senile plaques [64]. Δ^8^-THC also increased *DERL1*, encoding for Derlin-1, which plays a main role in the transport to the cytosol [65,66].

Δ^8^-THC also increased the expression of the shuttling factor *RAD23A* that delivers ubiquitin conjugates to the proteasome and activates its degradative capacity. Δ^8^-THC also restored the expression of other genes that were involved in substrate delivery to the proteasome, such as *ATXN3* [67].

Aβ_1-42_-treated cells also showed a downregulation of the genes encoding for 20S proteasome (*PSMA5*, *PSMB2*, *PSMB3*, *PSMB4*, *PSMB5*, *PSMB7*). The 20S proteasome was shown to be able to degrade misfolded, oxidized and intrinsically disordered proteins, but also Aβ, and to be the major degradation machinery under oxidizing conditions [68,69]. The 20S proteasome was reported to be inhibited in regions affected by Aβ, and Aβ aggregates were shown to inhibit proteasome activity in vitro [70]. It was shown that the Aβ precursor protein reduced the expression of the proteasome subunit α type-5 and β type-7, leading to cell death [71]. In line with the previous work, also in our work, these subunits were downregulated by Aβ_1-42_ treatment. Δ^8^-THC was able to partially upregulate the expression of proteasome subunits (*PSMB5*, *PSMB6*). In particular, we found the upregulation of *PSMB5,* whose overexpression was associated with increased resistance to Aβ_1-42_ toxicity [72].

As we also said before, ER stress can trigger neuronal apoptosis. The Aβ_1-42_-induction of cell death in our study was demonstrated by the increase in *BAX* and the reduction in *BCL2* gene expression. The transcriptomic results were also supported by Western blot analysis, which showed an increase in Bax protein levels and Bcl-2 reduction. Δ^8^-THC pre-treatment reduced apoptosis induced by Aβ_1-42_, as demonstrated by the increase in Bcl-2 and the reduction in Bax levels. 

RA-differentiated SH-SY5Y cells treated only with Δ^8^-THC showed no ER stress; indeed, *ATF6* and *ERN1* were not differentially expressed compared to the control cells, and *EIFAK3* was downregulated. Moreover, it increased some of the genes involved in the dislocation of misfolded proteins, such as *DERL1, VCP, SSR3* and some proteasome subunits, including *PSMB5,* suggesting an efficient degradation of potentially unfolded proteins. 

**Figure 5:**
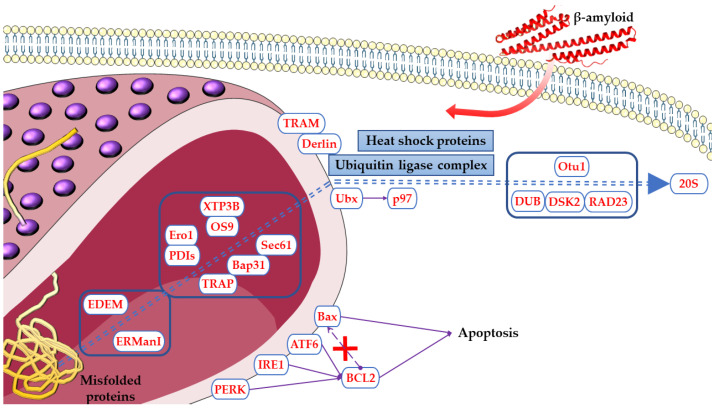
Proteins encoded by DEGs modulated in Aβ_1-42_ and Δ^8^-THC treated cells in ER pathway. The name of the shown proteins was obtained by KEGG. The figure was drawn using the vector image bank of Servier Medical Art by Servier (http://smart.servier.com/, accessed on 10 February 2023). Licensed under a Creative Commons Attribution 3.0 Unported License (https://creativecommons.org/licenses/by/3.0/, accessed on 10 February 2023).

Figure 5 reports the proteins encoded by DEGs and modulated in Aβ_1-42_ and Δ^8^-THC treated groups in the ER pathway.

## 4. Materials and Methods

### 4.1. Synthesis and Purification of Δ^8^-THC

To a stirred solution of CBD (200 mg, 0.636 mmol, 1eq) in DCM (5 mL), *p*-toluensulfonic acid (11 mg, 0.064 mmol, 0.1 eq) was added. The reaction was refluxed for 6 h, followed by TLC (R*f* = 0.67, silica, petroleum ether-EtOAc 95:5) until the complete conversion of the starting material, which was then quenched with NaHCO_3_ s.s. and diluted with DCM. The combined organic phases were washed with brine, dried, and evaporated. The residue was purified by GCC on silica gel (pure petroleum ether to petroleum ether-EtOAc 9:1) to afford 182 mg (91%) of Δ^8^-THC as a brown oil.

**Figure 6:**
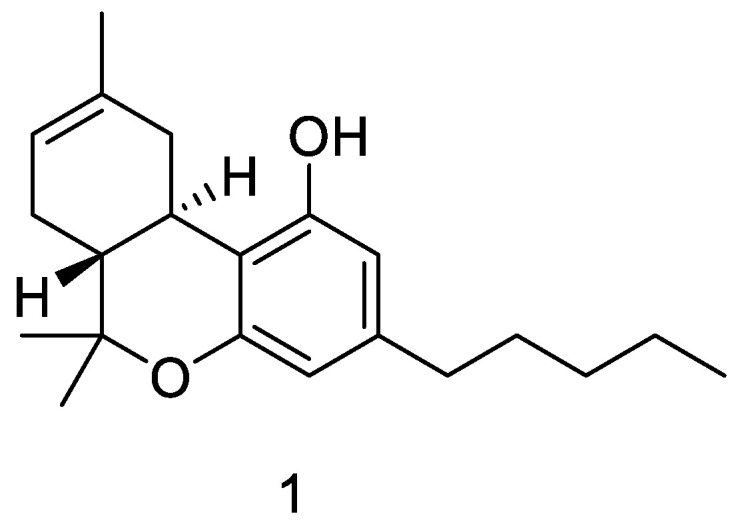
Δ^8^-THC chemical structure.

This latter impure Δ^8^-THC (1) (Figure 6) was purified with JASCO Hichrom, 250 × 25 mm, silica UV−vis detector-2075 plus (silica, petroleum-ether-EtOAc gradient from 95:5 to 85:15) to afford 150 mg of Δ^8^-THC (1, 99%) as a brownish powder, whose structure was identified according to ^1^H NMR (Figure S1) and reported in the literature [73]. ^1^H 400 MHz NM spectra were measured on Bruker 400 spectrometers (Bruker^®^, Billerica, MA, USA). Chemical shifts were referenced to the residual solvent signal (CDCl_3_: δH = 7.26). Silica gel 60 (70-230 mesh) used for low-pressure chromatography was purchased from Macherey-Nagel (Düren, Germany). Purifications were monitored by TLC on Merck 60 F254 (0.25 mm) plates, visualized by staining with 5% H_2_SO_4_ in EtOH and heating. Chemical reagents and solvents were from Aldrich (Darmstadt, Germany) and were used without any further purification unless stated otherwise. HCPL JASCO Hichrom, 250 × 25 mm, silica UV−vis detector-2075 plus (Tokyo, Japan).

### 4.2. Cell Culture and Differentiation

The human neuroblastoma cell line SH-SY5Y was acquired from American Type Culture Collection (ATCC) (Manassas, VA, USA). Cells were grown in a monolayer at 37 °C in a 5% CO_2_ humidified atmosphere using Dulbecco’s Modified Eagle’s Medium/Nutrient Mixture F-12 Ham (DMEM/F12) medium (Sigma-Aldrich, St. Louis, MO, USA) supplemented with 10% fetal bovine serum (FBS) (Sigma-Aldrich), 1% glutamine, and 1% penicillin-streptomycin (100 U-100 µg/mL). With the aim of inducing neuronal differentiation, SH-SY5Y cells were incubated for 5 days with 10 µM of RA (Sigma-Aldrich).

### 4.3. Cell Treatment with Aβ_1-42_ and Δ^8^-THC

Aβ_1-42_ (Sigma-Aldrich, St. Louis, MO, USA) was dissolved in dimethyl sulfoxide (DMSO), diluted in phosphate-buffered saline (PBS), aggregated at 37 °C for 24 h, and added to the medium at the concentration 10 µM (final DMSO concentration was <0.1%). It has been demonstrated that Aβ_1-42_ incubation for 24 h at 37 °C induced the formation of aggregates [74]. Δ^8^-THC was dissolved in DMSO, diluted in PBS and added at the final concentration in the medium (the final DMSO concentration was <0.1%). Cells were pre-treated with Δ^8^-THC for 24 h. At the end of the pre-treatment, cells were treated with the medium containing 10 µM of Aβ_1-42_ for 24 h. This concentration of Aβ_1-42_ was chosen based on previous studies showing that it was able to exert cytotoxicity in SH-SY5Y cells [75,76,77,78,79,80,81,82,83,84]. Control cells and cells pretreated with Δ^8^-THC were incubated with DMEM/F12 medium supplemented with 10% FBS. 

### 4.4. Cell Viability

Cell viability was evaluated with a Thiazolyl Blue Tetrazolium Bromide (MTT) assay. SH-SY5Y cells were cultured in 96-well plates, underwent RA differentiation, and were treated as reported in the previous paragraph. At the end of the treatment, the cells were incubated with a medium containing MTT (0.5 mg/mL; Sigma-Aldrich) at 37 °C for 4 h. The formed formazan crystals were dissolved in acidic isopropanol at 37 °C for 1 h, and the optical density was evaluated by the spectrophotometric measurement of absorbance using the microplate reader Victor NIVO^TM^ (PerkinElmer, Waltham, MA, USA). 

### 4.5. Extraction of Total RNA and cDNA Library Preparation

RNA extraction was carried out with a Maxwell^®^ RSC simplyRNA Cells Kit (Promega, Madison, WI, USA) according to the manufacturer’s instruction. The preparation of the library was performed following the TruSeq RNA Exome protocol (Illumina, San Diego, CA, USA) as previously described [75]. 

### 4.6. RNA-Seq Data Analysis and Gene Evaluation

The raw data obtained from the NextSeq 550 Dx instrument of Illumina was evaluated using the fastqc tool version 0.11.4 from the Babraham Institute in Cambridge, UK. Adapters and low-quality bases were then eliminated through Trimmomatic [85] version 0.38 (Usadel Lab, Aachen, Germany). The cleaned reads were aligned to the human reference genome (GRCh38) using the STAR RNA-seq aligner [86] 2.7.3a (New York, NY, USA). The expression levels of the transcripts were computed using the htseq-count python package [87] version 0.6.1p1 (European Molecular Biology Laboratory (EMBL), Heidelberg, Germany). DEGs were identified using the DESeq2 library in R [88] version 3.6.3 (R Core Team). No cut-off was set on the fold change. Nevertheless, to drop false positive DEGs, the Benjamini–Hochberg procedure was used with a tight q-value of 0.01. The enrichment of the biological process terms of the gene ontology was also performed in R using the package biomaRt [89] version 2.52.0. Plots were depicted using the R libraries ggplot2 version 3.4.0 and ggVennDiagram version 1.2.2.

### 4.7. Protein Extraction and Western Blot Analysis

At the end of the treatment, SH-SY5Y were harvested with trypsin-Ethylenediaminetetraacetic acid (EDTA), and proteins were extracted using RIPA (Thermo Scientific™, Waltham, MA, USA) according to the manufacturer’s instruction. Protein concentration was evaluated using the Bio-Rad Protein Assay (Bio-Rad Laboratories, Hercules, CA, USA) and bovine serum albumin (BSA) as standard. Twenty-five micrograms of proteins were separated on sodium dodecyl sulfate-polyacrylamide gel electrophoresis (SDS-PAGE) and transferred onto a PVDF transfer membrane (Immobilon-P PVDF, Merck Millipore division of Merck KGaA, Darmstadt, Germany). Membranes were blocked for 1 h at room temperature, with PBS containing 5% non-fat dried milk. Then, membranes were incubated with primary antibodies overnight at 4 °C. The following primary antibodies were used: Bax (1:1000; Cell Signaling Technology, Danvers, MA, USA) and Bcl-2 (1:1000; Cell Signaling Technology, Danvers, MA, USA). The membranes were incubated with secondary antibodies and horseradish peroxidase (HRP)-conjugated anti-rabbit IgG (1:2000; Santa Cruz Biotechnology, Inc., Dallas, TX, USA) for 1 h at room temperature. To evaluate that blots were loaded with equal amounts of protein lysates, they were also incubated with an antibody for GAPDH HRP Conjugated (1:1000; Cell Signaling Technology). The relative expression of protein bands was visualized using an enhanced chemiluminescence system (Luminata Western HRP Substrates, Millipore Corporation, Billerica, MA, USA), and protein bands were obtained and quantified with a ChemiDoc™ MP System (Bio-Rad Laboratories S.r.l., Hercules, CA, USA) and analyzed with the software Image J 1.54d. The uncropped blots for Bax and Bcl-2 and relatives of GAPDH are available in the supplementary Figures S2 and S3, respectively. 

### 4.8. Statistical Analysis

Statistical analysis of cell viability and Western blot was carried out using GraphPad Prism version 9.0 software (GraphPad Software, La Jolla, CA, USA). Multiple comparisons were performed using a one-way ANOVA test and the Bonferroni post hoc test. A *p*-value less than or equal to 0.05 was considered statistically significant. The results are expressed by the mean ± standard deviation (SD).

## 5. Conclusions

Δ^8^-THC reduced Aβ_1-42_-induced toxicity as a result of a reduction in ER stress. Indeed, Δ^8^-THC restored proteostasis, increasing the expression of proteasome and ubiquitin subunits and reducing UPR, suggesting that misfolded/unfolded proteins were not accumulated but could be eliminated through the proteasome. As a consequence of the reduced ER stress, Δ^8^-THC increased neuronal cell viability. The results suggested that Δ^8^-THC may represent a novel neuroprotective agent in AD but also in other neurodegenerative diseases characterized by the accumulation of misfolded proteins.

## Figures and Tables

**Table 1:** Common DEGs, altered in both CTRL vs. Aβ_1-42_ and Aβ_1-42_ vs. Δ^8^-THC *+* Aβ_1-42_, related to “Alzheimer’s disease” (hsa05010) and “protein processing in endoplasmic reticulum” (hsa04141) pathways.

Gene	CTRL vs. Aβ_1-42_		Aβ_1-42_ vs. Δ^8^-THC + Aβ_1-42_		CTRL vs. Δ^8^-THC	
	Fold Change	q-Value	Fold Change	q-Value	Fold Change	q-Value
*ATF6*	0.11	9.70 × 10^−7^	−0.11	9.51 × 10^−08^		
*BCL2*	−0.45	1.74 × 10^−17^	0.33	1.08 × 10^−10^	−0.34	3.16 × 10^−11^
*DNAJA2*	−0.11	8.15 × 10^−4^	0.10	1.40 × 10^−03^		
*DNAJC1*	0.21	1.51 × 10^−3^	−0.25	3.70 × 10^−05^		
*EDEM1*	−0.29	3.16 × 10^−9^	0.17	4.10 × 10^−04^		
*EIF2AK4*	0.15	1.51 × 10^−15^	−0.14	4.64 × 10^−16^	0.15	8.77 × 10^−15^
*ERN1*	0.37	1.46 × 10^−6^	−0.19	7.29 × 10^−03^		
*FBXO6*	−1.21	2.21 × 10^−4^	1.52	3.86 × 10^−07^		
*HSP90AA1*	0.06	3.31 × 10^−33^	0.01	5.68 × 10^−03^	0.11	3.57 × 10^−129^
*HSP90AB1*	0.04	2.31 × 10^−25^	−0.02	6.86 × 10^−05^	0.10	1.97 × 10^−145^
*HSP90B1*	0.06	2.20 × 10^−12^	−0.02	1.15 × 10^−03^	0.10	6.80 × 10^−43^
*MAN1B1*	0.10	1.70 × 10^−8^	−0.08	3.89 × 10^−06^		
*OS9*	0.16	2.08 × 10^−17^	−0.12	3.05 × 10^−12^		
*PSMB4*	−0.09	5.43 × 10^−13^	−0.06	4.45 × 10^−06^		
*PSMB5*	−0.18	3.86 × 10^−14^	0.14	2.98 × 10^−09^	0.09	2.45 × 10^−4^
*PSMB6*	0.14	1.82 × 10^−3^	0.15	7.90 × 10^−05^	0.21	1.16 × 10^−6^
*RAD23A*	−0.15	2.61 × 10^−6^	0.09	4.40 × 10^−03^		
*SEC61B*	0.12	1.06 × 10^−3^	−0.23	7.89 × 10^−13^		
*TXNDC5*	−4.20	2.95 × 10^−4^	3.22	8.52 × 10^−03^		
*UBE2G2*	−0.11	2.31 × 10^−3^	0.13	5.66 × 10^−05^		
*UBE2J1*	−0.11	7.60 × 10^−5^	0.11	6.06 × 10^−06^		
*UBXN6*	−0.11	4.66 × 10^−5^	0.11	1.41 × 10^−05^	0.08	2.60 × 10^−3^
*UBXN8*	−0.26	1.57 × 10^−5^	0.15	8.50 × 10^−03^		

The column fold change shows for each DEG the difference in the level of expression computed by log_2_(Aβ_1-42_/CTRL), log_2_(Δ^8^-THC + Aβ_1-42_/Aβ_1-42_) or log_2_(Δ^8^-THC/CTRL). The *q*-Value column was obtained correcting the *p*-value through Benjamini–Hochberg correction. All values were rounded to the second decimal digit.

## Data Availability

The data presented in this study are openly available in the NCBI Sequence Read Archive at BioProject accession numbers PRJNA934843.

## Supplementary Materials

The following supporting information can be downloaded at: https://www.mdpi.com/article/10.3390/ijms24076598/s1.
