# Supplementary material for: Δ8-THC Protects against Amyloid Beta Toxicity Modulating ER Stress In Vitro: A Transcriptomic Analysis
Source: Int J Mol Sci. 2023 Apr 2;24(7):6598. doi: 10.3390/ijms24076598 (PMC10095455; doi:10.3390/ijms24076598)
Supplement: Supplementary file 1 [file ijms-24-06598-s001.zip › ijms-2259364-supplementary.pdf]

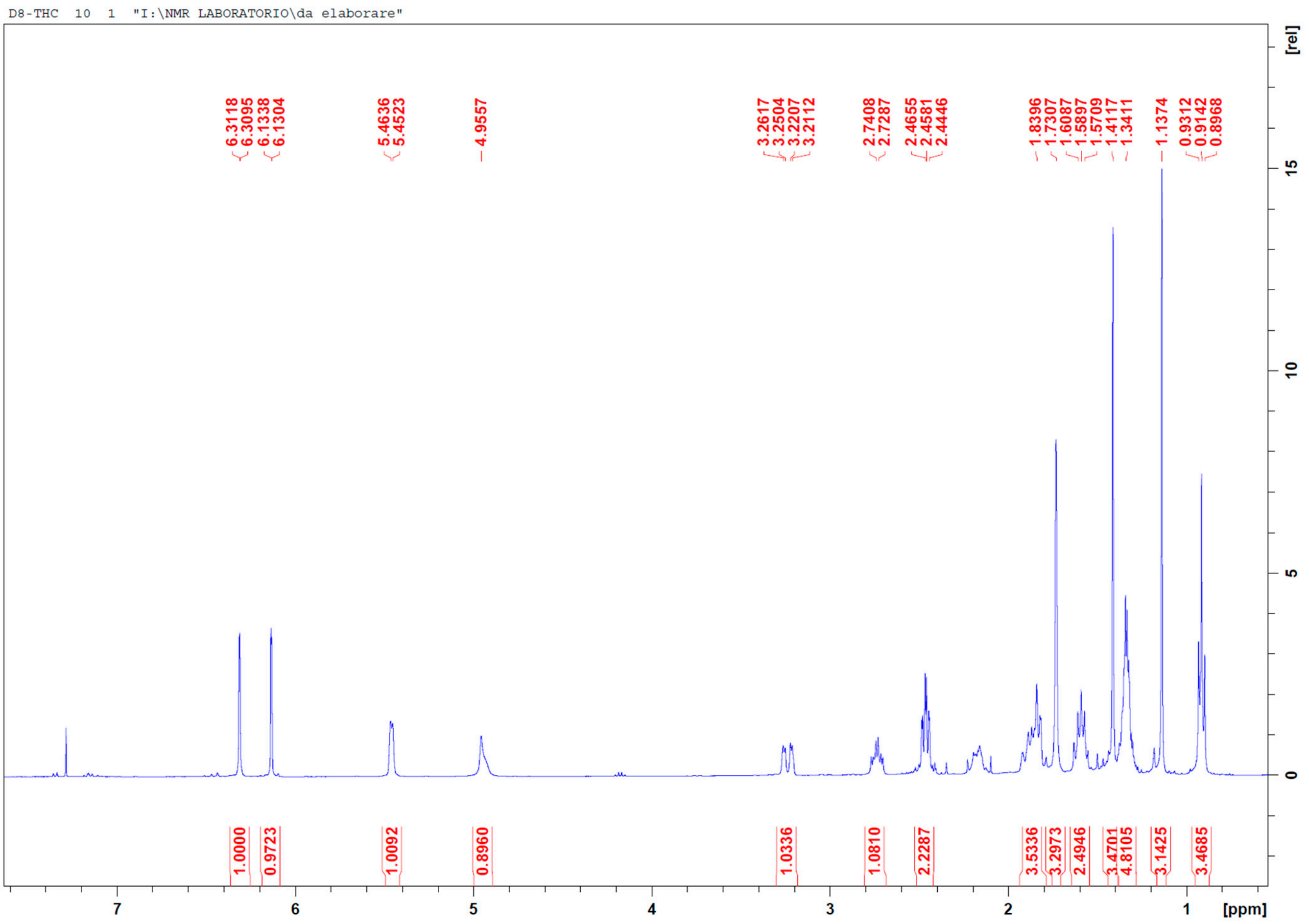

Figure S1: ^{1}H NMR of Δ^{8}-THC (1) in $CDCl_3$, 400 MHz.

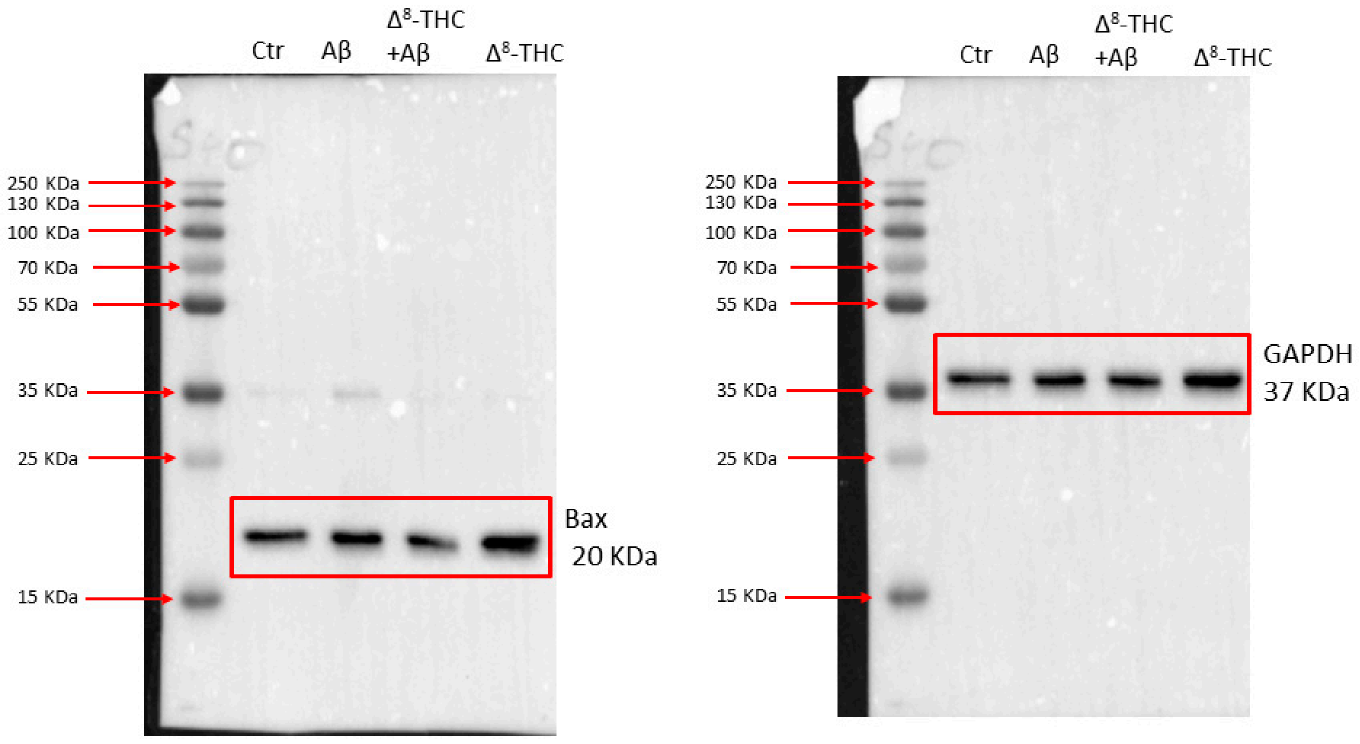

Figure S2: Uncropped blots for Bax and GAPDH.

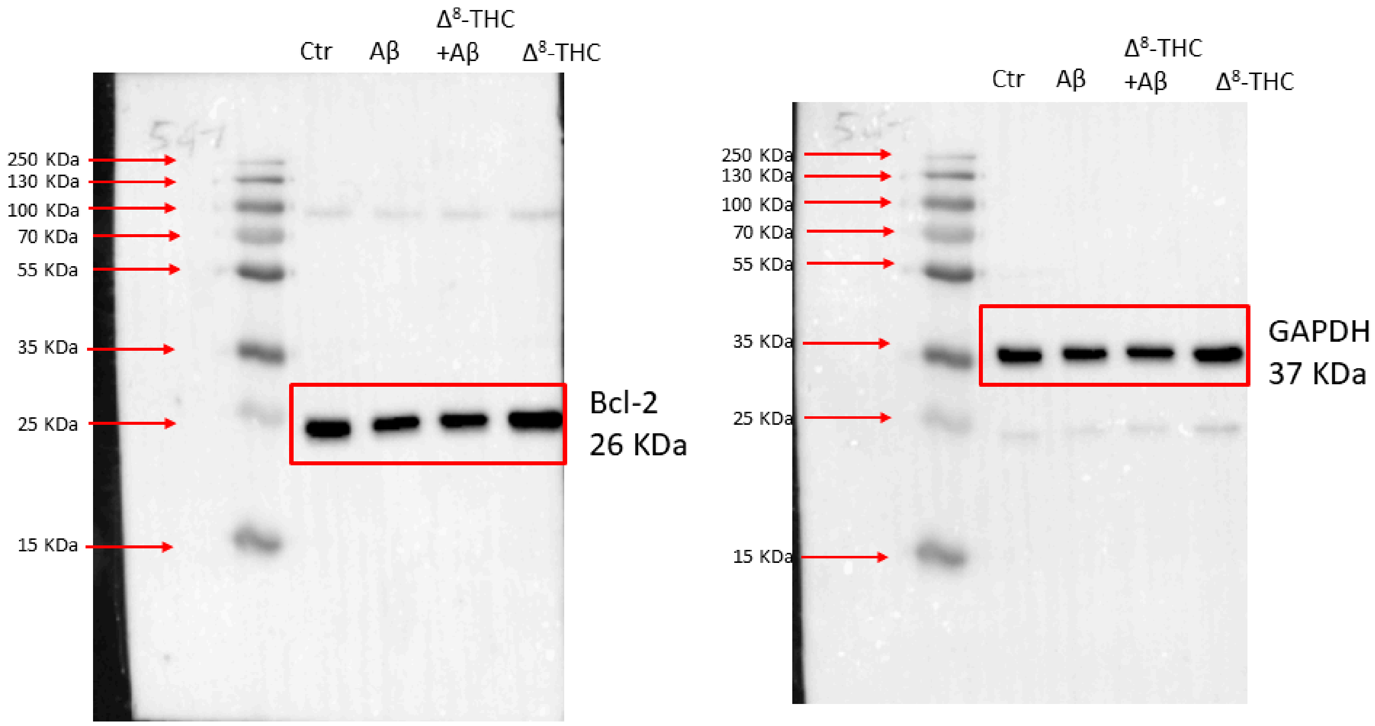

Figure S3: uncropped blots for Bcl-2 and GAPDH.

Table S1. Inspected DEGs involved in reticulum endoplasmic pathways.

Gene	CTRL	$A\beta_{1-42}$	Fold Change	q-Value	$A\beta_{1-42}$	Δ^8-THC + $A\beta_{1-42}$	Fold Change	q-Value	CTRL	Δ^8-THC	Fold Change	q-Value
ATF6	4264.87	4617.98	0.11	9.70×10^{-07}	5551.85	5127.2	-0.11	9.51×10^{-08}				
ATXN3					2029.33	2262.00	0.16	5.85×10^{-06}				
BAX	383.60	446.87	0.22	5.92×10^{-03}					410.44	489.50	0.25	1.08×10^{-03}
BCAP31	3644.71	3422.1	-0.09	7.17×10^{-04}								
BCL2	934.12	682.07	-0.45	1.74×10^{-17}	820.00	1032.61	0.33	1.08×10^{-10}	999.50	787.94	-0.34	3.16×10^{-11}
DERL1					1983.66	2227.06	0.17	1.52×10^{-06}	1713.93	1955.47	0.19	2.94×10^{-07}
DNAJA1	12806.77	14122.61	0.14	9.08×10^{-28}					13703.16	15581.19	0.19	1.29×10^{-50}
DNAJA2	2367.90	2190.94	-0.11	8.15×10^{-04}	2634.00	2823.82	0.10	1.40×10^{-03}				
DNAJB12	1169.58	1056.57	-0.15	2.36×10^{-03}					1251.44	1144.71	-0.13	6.89×10^{-03}
DNAJB2	610.21	705.59	0.21	8.48×10^{-04}								
DNAJC1	557.15	644.07	0.21	1.51×10^{-03}	774.32	651.93	-0.25	3.70×10^{-05}				
DNAJC10	8659.08	8985.39	0.05	1.83×10^{-03}					9265.16	9678.43	0.06	1.52×10^{-04}
DNAJC3	404.60	489.39	0.27	3.03×10^{-04}								
EDEM1	1122.04	920.88	-0.29	3.16×10^{-09}	1107.11	1244.10	0.17	4.10×10^{-04}				
EDEM3					3746.55	3337.83	-0.17	1.58×10^{-10}				
EIF2AK2	3973.03	4375.55	0.14	5.90×10^{-09}								
EIF2AK3					3202.78	3011.41	-0.09	2.58×10^{-03}	2771.39	2585.31	-0.10	1.46×10^{-03}
EIF2AK4	5794.83	6451.60	0.15	1.51×10^{-15}	7756.28	7018.64	-0.14	4.64×10^{-16}	6200.43	6869.93	0.15	8.77×10^{-15}
ERLEC1	3181.52	3426.62	0.11	1.06×10^{-04}					3404.20	3700.43	0.12	7.38×10^{-06}
ERN1	370.33	478.53	0.37	1.46×10^{-06}	575.30	503.89	-0.19	7.29×10^{-03}				
ERO1A	2105.90	2326.63	0.14	1.79×10^{-05}								
ERO1B					494.83	379.76	-0.38	4.80×10^{-07}	390.34	331.41	-0.24	6.41×10^{-03}
ERP29					2331.67	2516.71	0.11	9.32×10^{-04}	2059.32	2229.39	0.11	1.22×10^{-03}
FBXO6	37.59	16.28	-1.21	2.21×10^{-04}	19.58	56.09	1.52	3.86×10^{-07}				
HERPUD1					1232.17	1033.53	-0.25	5.56×10^{-08}				
HSP90AA1	89319.09	93064.28	0.06	3.31×10^{-33}	111884.22	112946.55	0.01	5.68×10^{-03}	95570.81	103369.33	0.11	3.57×10^{-129}
HSP90AB1	123032.36	126851.97	0.04	2.31×10^{-25}	152504.63	150772.56	-0.02	6.86×10^{-05}	131643.77	141335.70	0.10	1.97×10^{-145}

HSP90B1	36665.95	38100.82	0.06	2.20×10^{-12}	45805.77	45019.36	-0.02	1.15×10^{-03}	39232.32	42105.57	0.10	6.80×10^{-43}
HSPA1A	1291.18	1519.73	0.24	1.41×10^{-08}					1381.55	1777.93	0.36	8.12×10^{-21}
HSPA1L	135.97	173.68	0.35	7.61×10^{-03}								
MAN1A2	6096.62	6826.11	0.16	4.92×10^{-18}					6523.34	7281.65	0.16	8.48×10^{-18}
MAN1B1	7198.77	7720.76	0.10	1.70×10^{-08}	9282.09	8797.90	-0.08	3.89×10^{-06}				
MAN1C1	107.23	73.27	-0.55	1.26×10^{-03}					114.74	66.79	-0.78	3.95×10^{-06}
OS9	5930.80	6637.05	0.16	2.08×10^{-17}	7979.22	7327.60	-0.12	3.05×10^{-12}				
P4HB	11873.76	11509.22	-0.04	2.52×10^{-03}								
PDIA6	10631.23	9786.86	-0.12	1.46×10^{-15}								
PSMA5	2508.29	2180.08	-0.20	1.49×10^{-10}								
PSMB2	3642.50	3343.40	-0.12	2.85×10^{-06}					3897.45	3686.06	-0.08	2.46×10^{-03}
PSMB3	3379.40	3112.73	-0.12	1.68×10^{-05}								
PSMB4	14460.54	13561.75	-0.09	5.43×10^{-13}	16304.28	15663.91	-0.06	4.45×10^{-06}				
PSMB5	4152.11	3656.39	-0.18	3.86×10^{-14}	4395.80	4832.96	0.14	2.98×10^{-09}	4442.73	4721.70	0.09	2.45×10^{-04}
PSMB6	1235.91	1361.42	0.14	1.82×10^{-03}	1636.74	1820.64	0.15	7.90×10^{-05}	1322.41	1525.15	0.21	1.16×10^{-06}
PSMB7	5494.14	4641.50	-0.24	2.80×10^{-31}					5878.70	5640.68	-0.06	6.02×10^{-03}
RAD23A	2654.21	2399.00	-0.15	2.61×10^{-06}	2884.14	3061.98	0.09	4.40×10^{-03}				
RBX1	1266.86	1113.56	-0.19	4.67×10^{-05}								
SEC61A1					12847.02	12261.71	-0.07	2.07×10^{-06}	11466.42	11852.87	0.05	1.62×10^{-03}
SEC61B	1956.67	2121.29	0.12	1.06×10^{-03}	2550.26	2179.25	-0.23	7.89×10^{-13}				
SELENOS					1054.91	1186.17	0.17	5.35×10^{-04}				
SSR3					1028.81	1282.72	0.32	4.03×10^{-12}	1000.68	1130.33	0.18	4.44×10^{-04}
TRAM1					3012.46	3203.58	0.09	2.70×10^{-03}	2605.79	2860.07	0.13	1.21×10^{-05}
TXNDC5	16.58	0.90	-4.20	2.95×10^{-04}	1.09	10.11	3.22	8.52×10^{-03}				
UBE2G2	2112.54	1959.36	-0.11	2.31×10^{-03}	2355.59	2579.23	0.13	5.66×10^{-05}				
UBE2J1	3562.90	3309.02	-0.11	7.60×10^{-05}	3978.19	4300.56	0.11	6.06×10^{-06}				
UBQLN1	7563.57	6966.32	-0.12	3.39×10^{-11}					8092.97	7629.97	-0.08	1.92×10^{-06}
UBQLN2	6254.70	5359.75	-0.22	4.34×10^{-30}					6692.49	5874.86	-0.19	5.37×10^{-23}
UBQLN4	3088.66	2724.65	-0.18	1.79×10^{-10}					3304.84	2804.28	-0.24	6.67×10^{-18}
UBXN6	3534.16	3274.65	-0.11	4.66×10^{-05}	3936.87	4245.39	0.11	1.41×10^{-05}	3781.53	3995.48	0.08	2.60×10^{-03}
UBXN8	762.77	638.65	-0.26	1.57×10^{-05}	767.80	854.23	0.15	8.50×10^{-03}				

VCP	23353.98	22050.52	-0.08	1.34×10^{-16}					24988.60	25470.98	0.03	8.05×10^{-03}
WFS1	1678.09	1444.65	-0.22	3.16×10^{-08}								
YOD1	517.36	442.35	-0.23	2.07×10^{-03}					553.57	481.05	-0.20	4.99×10^{-03}

The column Fold Change shows for each DEG the difference in the level of expression computed by $\log_2(A\beta_{1\text{-}42}/CTRL)$, $\log_2(\Delta^8\text{-THC} + A\beta_{1\text{-}42}/A\beta_{1\text{-}42})$ or $\log_2(\Delta^8\text{-THC}/CTRL)$. The *q*-Value column was obtained correcting the p-value through Benjamini–Hochberg correction. Blank cells are shown when gene q-value is higher than 0.01.
